# Clinical Outcomes of Single Incision Laparoscopic Cholecystectomy in the Anglophone Caribbean: A Multi Centre Audit of Regional Hospitals

**Published:** 2014-09

**Authors:** Shamir O. Cawich, Matthew Albert, Yardesh Singh, Dilip Dan, Sanjib Mohanty, Maurice Walrond, Wesley Francis, Lindberg K. Simpson, Kimon O. Bonadie, Giovanni Dapri

**Affiliations:** 1 Department of Surgery, University of the West Indies, St. Augustine Campus, Trinidad & Tobago;; 2 Department of Surgery, Florida State University, Tallahassee, Florida, USA;; 3 Department of Surgery, Cayman Islands Hospital, Grand Cayman, UK;; 4 Department of Surgery, University of the West Indies, Cave Hill Campus, Barbados;; 5 Department of Surgery, Princess Margaret Hosptial, Bahamas;; 6 Department of Gastrointestinal Surgery, European School of Laparoscopic Surgery, Saint-Pierre University Hospital, Brussels, Belgium

**Keywords:** Cholecystectomy, Caribbean, Single Incision, Laparoscopic, Gallbladder

## Abstract

**Introduction:**

There has been no report on Single-Incision Laparoscopic Surgery (SILS) cholecystectomy outcomes since it was first performed in the Anglophone Caribbean in 2009.

**Methods:**

A retrospective audit evaluated the clinical outcomes of SILS cholecystectomies at regional hospitals in the 17 Anglophone Caribbean countries. Any cholecystectomy using a laparoscopic approach in which all instruments were passed through one access incision was considered a SILS cholecystectomy. The following data were collected: patient demographics, indications for operation, intraoperative details, surgeon details, surgical techniques, specialized equipment, conversions, morbidity and mortality. Descriptive statistics were generated using SPSS 12.0.

**Results:**

There were 85 SILS cholecystectomies in women at a mean age of 37.4 ± 8.5 years with a mean BMI of 30.9 ± 2.8. There were 59 elective and 26 emergent cases. Specialized access platforms were used in the first 35 cases and reusable instruments were passed directly across fascia in the latter 50 cases. The mean operative time was 62.9 ± 17.9 minutes. There was no mortality, 2 conversions to multi-trocar laparoscopy and 5 minor complications. Ambulatory procedures were performed in 43/71 (60.6%) patients scheduled for elective operations.

**Conclusion:**

In the Caribbean setting, SILS cholecystectomy is a feasible and safe alternative to conventional multi-trocar laparoscopic cholecystectomy for gallbladder disease.

## INTRODUCTION

While “conventional” laparoscopic surgery undoubtedly decreases surgical morbidity, it requires 3 or 4 incisions, each with a potential for pain, bleeding, inter-fascial hematoma, visceral injury, local nerve irritation, incisional hernia formation and compromised cosmesis (1, 2). Surgeons recognized this in the late 1900s and began to develop techniques to further reduce surgical trauma such as single incision laparoscopic surgery (SILS), natural orifice trans-luminal endoscopic surgery (NOTES) and needlescopic surgery.

In the Anglophone Caribbean, conventional multi-trocar laparoscopic cholecystectomy was first performed in Trinidad and Tobago in 1991 (3). In the subsequent two decades, abundant data were accrued on the outcomes of conventional laparoscopic cholecystectomy (3-12). However, there has been no documentation of outcomes with SILS cholecystectomy since it was first performed in the Caribbean in 2009 (13). We performed a retrospective audit to evaluate the clinical outcomes of SILS cholecystectomies at regional hospitals across the Anglophone Caribbean.

## MATERIALS AND METHODS

The Anglophone Caribbean includes all the independent English-speaking countries of the Caribbean (14): Antigua and Barbuda, the Bahamas, Barbados, Dominica, Grenada, Jamaica, St. Kitts and Nevis, St. Lucia, St. Vincent and the Grenadines, Trinidad and Tobago, Guyana and Belize. The current Caribbean British overseas territories were also included in this audit: Anguilla, British Virgin Islands, Cayman Islands, Montserrat and Turks and Caicos. These 17 countries have a cumulative estimated population of 6,426,914 persons (15).

The University of the West Indies was founded in 1948 to serve as a regional medical institution supported by and serving these 17 Caribbean countries (11). Therefore, ethical approval for this study was sought from and granted by the University of West Indies’ review board.

Investigators performed a survey of surgeons in each territory and retrospectively examined records from operating theatres in each of these countries from January 1, 2009 to January 30, 2013. The records for patients who had SILS cholecystectomy were retrieved. Data were extracted and entered in a Microsoft Excel worksheet. The information collected included patient demographics, indications for operation, intraoperative details, surgeon details, surgical techniques, specialized equipment, conversions, morbidity and mortality. Descriptive statistics were generated using SPSS 12.0.

Any cholecystectomy using a laparoscopic approach in which all instruments and laparoscopes were passed through a single access incision was considered a SILS cholecystectomy. A conversion was considered to be any procedure in which an additional incision was required separate from the umbilical incision - whether for open access, to place an additional port or introduce other devices to assist in the exposure of the Calots’ triangle.

Any cholecystectomy performed in an operating room on anesthetized patients requiring <24 hours hospitalization was considered an ambulatory procedure. This is the standardized definition used by the United States Planning and Research Cooperative System committee (16).

Complications were graded according to their severity using the standardized grading system for surgical complications proposed by Clavien (17). Grade I-II complications were considered mild and grade III-IV complications as major morbidity (17). Post-operative mortality was defined as death from any cause within 30 days of operation.

## RESULTS

Over the study period, there were 85 SILS cholecystectomies performed across the region. The technique was utilized in Jamaica [45], Cayman Islands [8] and Trinidad & Tobago [32] as outlined in Table 1.

**Table 1:** Outcomes of SILS cholecystectomy in the Caribbean by territory

Parameter	Jamaica	Trinidad & Tobago	Cayman Islands

Number of cases	45	32	8
Morbidity	3	1	2
Mortality	0	0	0
Operating time	62.4 ± 18.5	59.9 ± 15.0 mins	77.8 ± 20.4 min
Hospitalization	18.3 ± 28.3 Hrs	39.25 ± 37.5 Hrs	76.0 ± 39.4 Hrs

These cases were all performed in females at a mean age of 37.4 years (SD ± 8.5; Range 18-60) and a mean body mass index of 30.9 ± 2.8 (SD) (Range 24-36). There were 59 (69.4%) elective procedures performed for biliary colic [4] and chronic cholecystitis [55] and 26 (30.6%) were performed emergently for acute cholecystitits.

Peritoneal access was always attained using an open Hasson’s technique at the umbilicus, but there were many access platforms in use. Most cases [25] were performed using the Covidien SILS port ® (Covidien, Inc., Norwalk, CT, USA). In the other cases, peritoneal access was achieved with the Gelpoint Access Platform (Applied Medical, Rancho Santa Margarita, CA, USA) in 8 cases and the InnoPort ® (Innovia LLC, Miami, FL, USA) in 2 cases. Standard 35cm straight laparoscopic instruments and conventional laparoscopes were used in these 35 cases.

**Figure 1:**
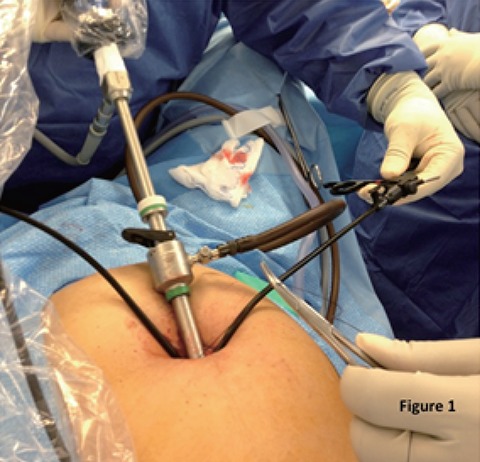
SILS technique using a single 11 mm trocar (visual) with curved reusable instruments passed directly across the fascia without access ports.

In the latter 50 cases specialized access ports were abandoned in favour of a previously described technique (18) using a single reusable 11 mm standard trocar with 5 mm DAPRI curved reusable instruments (Karl Storz Endoskope, Tuttlingen, Germany) passed directly across the fascia (Fig. 1). Regardless of the access platform utilized, similar techniques were used for intra-corporal dissection and identification of biliary structures. No operative cholangiograms or additional procedures were performed in this series.

The operations were completed in an average time of 62.9 minutes (SD ± 17.9; Range 45-90; Mode 70; Median 60). There were 2 (2.4%) conversions where supplemental trocars were required. The average duration of hospitalization for all patients was 31.6 hours (SD ± 37.0; range 4-144; median 10; mode 48). Ambulatory procedures were performed in 43/71 (60.6%) patients scheduled for elective operations.

**Figure 2:**
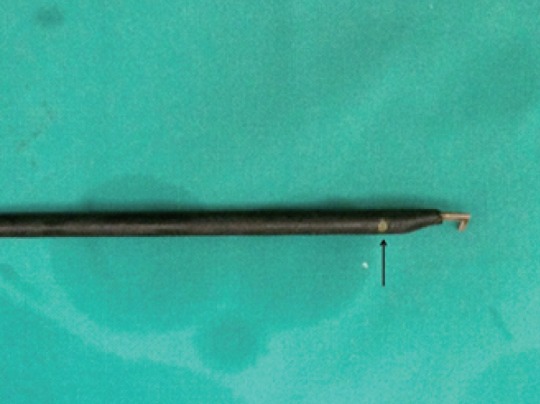
Sheared insulation (arrow) near the tip of an electrocautery hook allowing lateral discharge of energy during dissection.

There was no mortality in this series. There were minor complications in 5 (5.9%) cases: 3 wound infections (Grade 1), 1 diaphragmatic laceration repaired with intra-corporeal sutures without conversion (Grade 2a) and 1 bile leak (Grade 2b). The bile leak occurred in a 45 year old woman who had multiple prior attacks of acute cholecystitis. Intra-operatively, a retrograde technique was used with a 30° rigid laparoscope and standard straight instrumentation. During the procedure, it was noted that the electrocautery hook was exposed due to shearing of the insulation near the instrument tip (Fig. 2). The instrument was immediately changed but bile was seen leaking from a common duct injury occupying 15-20% of the duct circumference - presumably from lateral discharge of energy during dissection in Calot’s triangle. A supplemental 5mm trocar was used to intubate the injury laparoscopically and the T-tube was brought through the trocar skin incision. This allowed adequate healing without the need for any additional procedures after 32 months of follow-up.

**Figure 3:**
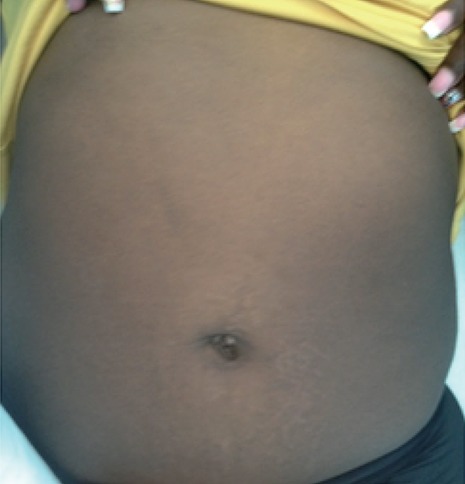
Post-operative picture of a patient’s abdomen 12 weeks after a successful SILS cholecystectomy.

All patients who had SILS cholecystectomy completed expressed satisfaction with the cosmetic outcomes, generally noting that their scar was not appreciable at their 6 week clinic visits (Fig. 3).

## DISCUSSION

Navarra *et al*. reported the first SILS cholecystectomy in 1997 in Italy (19). The first case in the Caribbean was performed over a decade after its original description (13). A major obstacle to the earlier adoption of this technique was the cost associated with specialized SILS equipment (20).

Since Navarra’s original description (19), many published studies have documented outcomes of SILS cholecystectomies using several different techniques and access platforms. There have been few small randomized-controlled trials comparing SILS and conventional multiport techniques (21-26). Although the results are mixed, most have documented that SILS brings greater costs (25) and longer operating times (21, 26) but similar duration hospitalization (21, 22) and similar complication rates (21, 22). Some have also documented better patient satisfaction (25, 26), quality of life (25), cosmesis (21-26) and post-operative pain scores (22-26) with SILS compared to multiport laparoscopy.

Our preliminary results suggest that SILS cholecystectomy can also be performed safely in this setting. The overall morbidity in this series (5.9%) was comparable to reports of conventional laparoscopic cholecystectomy within the Caribbean, where overall morbidity ranges from 1.5% (3) to 8% (4). They were also comparable to the published randomized trials where overall morbidity ranged from 0 (24) to 9.5% (21).

Opponents originally suggested that the demand for operating time would be increased with the introduction of a new technique (27), effectively resulting in an increase in our case backlog. This would have a direct impact on the region as it has been previously documented that up to 18% of patients having open cholecystectomy cited long waiting lists for laparoscopic surgery as their reason to choose the open approach (9). However, we have demonstrated that the operating time required for SILS cholecystectomy (62.9 minutes) is comparable to that in reports of conventional laparoscopic cholecystectomy from the region, ranging from 34 (3) to 83 minutes (9). The operating time is also comparable to that documented in other countries where SILS operating times ranged from 43.5 (24) to 88.5 minutes (21) in the existing randomized trials.

It is well established that ambulatory laparoscopic cholecystectomy can be performed safely, while significantly reducing the associated cost (28). However, there have been very low rates of ambulatory laparoscopic cholecystectomy documented across Caribbean territories, ranging from 40% (9) to 52% (3) of cases. Several reasons have been suggested, but foremost has been the “cultural” resistance by patients who expect to be hospitalized after an abdominal operation regardless of the approach (21). The rate of elective ambulatory procedures with SILS is higher than all reports of conventional laparoscopic cholecystectomies originating from the Caribbean (2-12). Our experience has been that the patients’ expectations to remain hospitalized are tempered once they realize that the operations are completed and they are left with a single small incision. This may be one reason more patients have been accepting of ambulatory procedures in this report.

Compared to conventional multitrocar laparoscopic cholecystectomy, there should no difference in cost to perform a SILS cholecystectomy since we use reusable instruments without any specialized visual systems or access ports. Additionally, the curved reusable instruments allow effective triangulation, while minimizing instrument collision with good surgeon ergonomics. Other maneuvers that we have found beneficial and routinely employ include the use of a 30° bariatric length laparoscope, right angle light lead and an experienced and vigilant camera controller.

Even with these maneuvers, SILS cholecystectomy can be technically challenging. Although the curved instruments allow less instrument collision, they still require the surgeon to perform counterintuitive tasks with a restriction on the freedom of instrument movement since all instruments fulcrum through one entry point. Therefore, surgeons must have advanced laparoscopic experience and training in SILS procedures, before they embark on these techniques (29).

## CONCLUSIONS

In the Caribbean setting, SILS cholecystectomy is a feasible and safe alternative to conventional multi-trocar laparoscopic cholecystectomy for gallbladder disease.

## References

[1] Lowry PS, Moon TD, D’AlessandroA A, Nakada SY (2003). Symptomatic port-site hernia associated with a non-bladed trocar after laparoscopic live-donor nephrectomy. J. Endourol.

[2] Marcovici I (2001). Significant abdominal wall hematoma from an umbilical port insertion. JSLS.

[3] Dan D, Harnanan D, Maharaj R, Seetahal S (2009). Lapaoroscopic Cholecystectomy: An Analysis of 619 consecutive cases in a Caribbean Setting. J. Natl. Med. Assoc.

[4] Mitchell DIG, DuQuesnay DR, McCartney T, Bhoorasingh P (1996). Laparoscopic cholecystectomy in Jamaica. West Ind. Med. J.

[5] McFarlane ME, Thomas C, McCartney T, Bhoorasingh P (2003). Laparoscopic Cholecystectomy Without Routine Intra-Operative Cholangiograms: A Review of 136 Cases in Jamaica. West Ind. Med. J.

[6] Bailey HH, Dan DV (2005). An Economic Evaluation of LC for public hospitals in Trinidad and Tobago. West Ind. Med. J.

[7] McFarlane ME, Thomas C, McCartney T, Bhoorasingh P (2005). Selective Operative Cholangiography in the Performance of Laparoscopic Cholecystectomy. Int. J. Clin. Pract.

[8] Plummer J, Duncan N, Mitchell D, McDonald A (2006). Laparoscopic cholecystectomy for chronic cholecystitis in Jamaican patients with sickle cell disease: preliminary experience. West Ind. Med. J.

[9] Cawich SO, Mitchell DIG, Newnham MS, Arthurs M (2006). A Comparison of Open and Laparoscopic Cholecystectomy by a Surgeon in Training. West Ind. Med. J.

[10] Cawich SO, Mathew AT, Mohanty SK, Huizinga WK (2008). Laparoscopic Cholecystectomy: A Retrospective Audit from The Cayman Islands. Int. J. Surg.

[11] Plummer JM, Roberts PO, Leake PA, Mitchell DIG (2011). Surgical care in Jamaica in the laparoendoscopic era: challenges and future prospects for developing nations. Perm. J.

[12] Dan D, Seetahal S, Harnanan D, Singh Y (2009). Laparoscopic cholecystectomy in Sickle cell disease patients: Does operating time matter?. International J. Surg.

[13] Cawich SO, Albert M, Mohanty SK, Dapri G (2012). Laparoscopic Cholecystectomy with Straight Instruments Through One Incision: Learning from The Early Experience in Jamaica. Int. J. Surg.

[14] Commonwealth Caribbean Library of Congress. USA 1989. http://lcweb2.loc.gov/frd/cs/Caribbeanislands/cxappnc.html.

[15] 15. Wikipedia (2010) Geography; Demographics / Countries by Population. http://en.wikipedia.org/wiki/List_of_countries_by_population.

[16] De Jong JD, Westert GP, Lagoe R, Groenewegen PP (2006). Variation in hospital length of stay: Do physicians adapt their length of stay decisions to what is usual in the hospital in which they work?. Health Serv. Rev.

[17] Clavien PA, Sanabria JR, Strasberg SM (1992). Proposed classification of complications of surgery with examples of utility in cholecystectomy. Surg.

[18] Dapri G, Casali L, Dumont H, VanderGoot L (2011). Single access trans-umbilical laparoscopic appendectomy and cholecystectomy using new curved reusable instruments: A pilot feasibility study. Surg. Endoscopy.

[19] Navarra G, Pozza E, Occhionorelli S, Carcoforo P (1997). One-wound laparoscopic cholecystectomy. Br. J. Surg.

[20] Dan D, Naraynsingh V, Cawich SO, Jonnagoladda R (2012). The History of Laparoscopic General Surgery in the Caribbean. West Ind. Med. J.

[21] Ma J, Cassera M, Spaun G, Hammill C (2011). Randomized Controlled Trial Comparing Single-Port Laparoscopic Cholecystectomy and Four-Port Laparoscopic Cholecystectomy. Ann. Surg.

[22] Asakuma M, Hayashi M, Komeda K, Shimizu T (2011). Impact of single-port cholecystectomy on postoperative pain. Br. J. Surg.

[23] Tsimoyiannis EC, Tsimogiannis KE, Pappas-Gogos G, Farantos C (2010). Different pain scores in single trans-umbilical incision laparoscopic cholecystectomy versus classic laparoscopic cholecystectomy: a randomized controlled trial. Surg. Endosc.

[24] Lai EC, Yang GP, Tang CN, Yih PC (2011). Prospective randomized comparative study of single incision laparoscopic cholecystectomy versus conventional four-port laparoscopic cholecystectomy. Am. J. Surg.

[25] Bucher P, Pugin F, Buchs NC, Ostermann S (2011). Randomized clinical trial of laparo-endoscopic single-site versus conventional laparoscopic cholecystectomy. Br. J. Surg.

[26] Lee PC, Lai PS, Chang JJ, Huang SJ (2010). Randomized clinical trial of single-incision laparoscopic cholecystectomy versus mini-laparoscopic cholecystectomy. Br. J. Surg.

[27] Cawich SO, Cherian CJ, Wilson C, Baker A (2012). Challenges against the advancement of minimally invasive surgery in Jamaica: A national hospital survey. West Ind. Med. J.

[28] Lau H, Brooks DC (2002). Contemporary outcomes of ambulatory laparoscopic cholecystectomy. WJS.

[29] Raman JD, Bensalah K, Bagrodia A, Stern JM (2007). Laboratory and clinical development of single keyhole umbilical nephrectomy. Urology.

